# Metastatic tumours to the thyroid gland: report of 3 cases and brief review of the literature

**DOI:** 10.2478/v10019-010-0039-3

**Published:** 2010-09-22

**Authors:** Enver Vardar, Nazif Erkan, Umit Bayol, Cengiz Yılmaz, Murat Dogan

**Affiliations:** 1 SB Izmir Tepecik Teaching Research Hospital, Pathology Department, Turkey; 2 SB Izmir Bozyaka Teaching Research Hospital, Surgery Department, Turkey; 3 SB Izmir Bozyaka Teaching Research Hospital, Radiology Department, Turkey

**Keywords:** metastatic tumours, thyroid malignancy, secondary malignancies

## Abstract

**Background:**

Metastases to the thyroid are encountered rarely in clinical practice, but the number of cases seems to have increased in recent years. The reason of this increase may be a more frequent use of fine-needle aspiration biopsy (FNAB) and the use of more sophisticated, complicated imaging techniques in patients with thyroid masses. Also, in addition to these reasons, the use of more organo-specific immunohistochemical antibodies in the examination of surgical specimens may affect the differential diagnosis of malignant tumours.

**Case reports:**

Three metastatic tumours to thyroid were found in the retrospective review of malignant thyroid tumours diagnosed between January 1993 and December 2007. The primary tumours were clear cell carcinoma of the kidney, squamous cell carcinoma of the lung and breast carcinoma-ductal type.

**Conclusions:**

A detailed clinical history, careful histological examination and essential immunohistochemistry helped in attaining the correct diagnosis.

## Introduction

Despite being 2^nd^ only to the adrenal gland in the relative vascularisation, the thyroid gland is rarely the site of the significant metastatic disease.^1^ Metastatic tumours to the thyroid are seen rarely in clinical practice, but the incidence varies from 1.25% to 24.2% in autopsy series.^2^–^8^ In autopsy series, the metastasis is usually associated with the vascular tumour embolus from tumour located in distant organ malignancy or with the direct invasion of the thyroid by a malignant focus located in the adjacent organs.^5^–^8^ Most commonly, the primary tumour is located in the lung, gastrointestinal system, breast and kidney. Secondary tumours of the thyroid are seen almost always with metastases of other organs and systems. The surgical resection was rarely performed in the metastasis to the thyroid gland due to the presence of the phenomenon of secondary tumours of the thyroid which is rarely discernible clinically. And because of this, the metastasis to the thyroid gland is rarely seen on the routine pathology practice. The metastasis to the thyroid gland is usually considered as a terminal event, and the effectiveness of the conventional treatment has been questioned.^2^–^4^,^7^ Sporadic reports and small case series were published in the literature. We report three cases of metastatic malignancy to the thyroid with the review of the literature.

## Case reports

### Case 1

A 54-year-old Caucasian male presented to the outpatient clinic with the enlargement on the right thyroid lobe noted approximately 2 months earlier. In his medical history he was operated due to clear cell carcinoma of the right kidney three years ago. The pathological examination of the kidney was revealed as pT3N1M0, Grade 2. Then the adjuvant chemotherapy and the interferon treatment were planned but he refused any treatment and he has been followed after the operation by routine examinations such as liver functions (SGOT, SGPT, alkaline phosphatase, GGT, bilirubin levels), urea, creatinine levels, abdominal ultrasonography, bone scintigraphy, chest X-ray.

During that follow up period, the patient had no symptoms and routine tests were within normal limits. The physical examination after three years revealed solitary nodule on the right lobe of the thyroid that were thought to be an adenoma or a nodule of colloidal goitre. A fine needle aspiration biopsy of the thyroid revealed some mildly pleomorphic and hyperchromatic cells and due to this suspicious ctyological finding consistent with malignancy, bilateral near total thyroidectomy was performed.

The pathological specimen measured 5.5 × 5 × 3 cm and showed two nodules, each of them located at different lobe and measured up to 2.0 cm in the greatest dimension. A microscopic examination showed the diffuse proliferation of acinary structures and large sheets of neoplastic cells in the adjacent acini with the extension into the thyroid follicular tissue. The neoplastic cells had a moderate amount of clear, vacuolar or mildly eosinophilic granular cytoplasm and pleomorphic, hyperchromatic nuclei with large nucleoli. Many of the cells showed multinucleation, nuclear lobulation and high mitotic activity, as shown on immunohistochemical staining of tumour tissue sections (Figure 1A). CD10 (Figure 1B), vimentin and pancytokeratin positivity, however, in the same tumour areas cells were negative with TTF-1 (Figure 1C), thyroglobulin and calcitonin.

The patient was uneventful after the operation, and the adjuvant chemotherapy treatment for the metastatic disease was planned, however, he again refused chemotherapy. Eighteen months after the thyroid surgery, he was admitted to our emergency service with right hemiplegia. Cranial CT revealed a metastatic tumour and he was operated for brain metastases. During operation, the metastasis was removed successfully but he died due to pulmonary embolia 5 days after the operation.

### Case 2

A 63-year-old Caucasian male presented with a growing thyroid mass over the past 2 months. The clinical examination showed the enlarged right lobe of the thyroid. Ultrasonography and computed tomography (CT) scan of the thyroid revealed a growing single nodule in the right lobe and the diameter of the nodule measured 1.4 cm. The patient was operated due to the presence of lung malignancy eleven months before and diagnosed as primary squamous cell carcinoma of the right lung. After right lobectomy, pathology was reported as pT2N2M0, Stage 3A. The central nervous system, bilateral neck, liver and spleen were normal. Adjuvant chemotherapy that consisted of cisplatin and etoposide regimen was given postoperatively.

**FIGURE 2.**
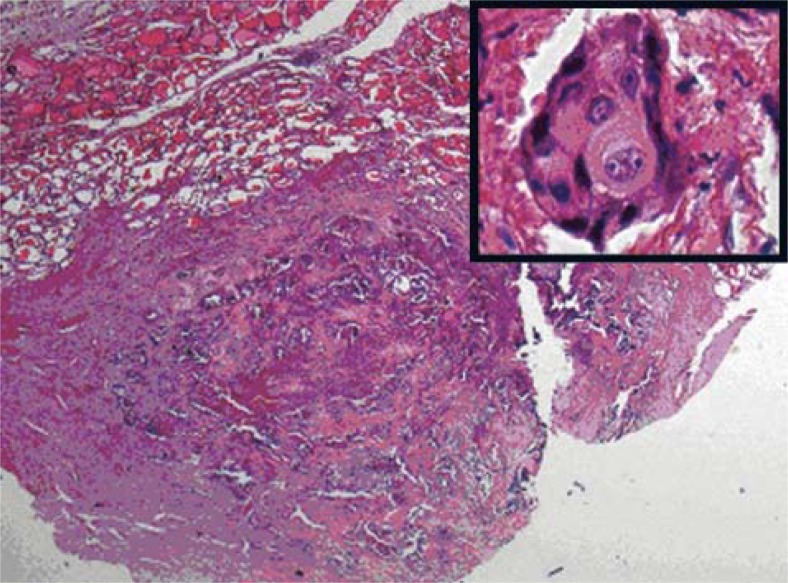
Large, solid tumour island contain necrosis in central portion were seen in above of the image. Residual normal thyroid follicles are present in left upper part of the image (HE-X20). Squamous differentiation consistent with acidophilic cytoplasm, vesicular nuclei and intercellular bridges were seen in tumour cells (inset) (HE-X400).

The fine needle aspiration biopsy of the thyroid right lobe of the patient demonstrated distinct malignant cytologic findings and after that total thyroidectomy was performed and sent for a histopathological examination. The microscopic examination showed thyroid tissue partially replaced by a neoplastic infiltrate composed of pleomorphic cells in bilateral lobe in addition to nodule located in the right lobe of the thyroid. Some of the cells have intercellular desmosomes and keratinisation that the hallmark of squamous cell carcinoma (Figure 2). Desmoplastic stroma was seen mainly in between squamous cell carcinoma areas. TTF-1, thyroglobulin, calcitonin, CK20 and CK7 were negative in metastatic tumour areas.

The patient died after 6 months due to disseminated metastases.

### Case 3

A 43-year-old female was admitted with complaints of a mass in the thyroid for the past 2 months. The clinical examination showed the enlarged bilateral thyroid gland. An ultrasonography of the thyroid revealed diffuse enlargement and multinodular pattern. Left modified radical mastectomy and left axillary dissection were performed due to the presence of the malignant tumour in her left breast. Pathology was reported as pT2N2M0 and stage 3A invasive ductal carcinoma.

Radiotherapy and chemotherapy were applied and regular follow-ups were done for three years. Bilateral total thyroidectomy was performed and no problem was seen in the surgery of thyroid. In addition to the nodular appearance of cut surface, multiple, tiny whitish-solid areas were seen. In microscopic evaluation, findings consistent with colloidal goitre were seen. Also, between nodular structures of thyroid acini, small solid tumour islands were present (Figure 3A). Pleomorphic, hyperchromatic nuclei with mitotic figures were present in these tumour sheets. There were no specific findings except abortive glandular appearance. Immunohistochemically, tumour cells were stained with CK7 and focally with ER and metastatic tumour cells were negative with, progesterone, cerbB-2, TTF-1 (Figure 3B), thyroglobulin, CEA and calcitonin.

After the operation for the metastatic disease, the adjuvant chemotherapy was planned but she refused that treatment. The patient died after 22 months due to the disseminated metastases in liver and bone.

Since there are no specific tumour markers for squamous cell cancer of the lung, clear cell cancer of the kidney and invasive ductal cancer of the breast, we did not use any tumour marker for the detection of metastases or local recurrence of the disease during the follow-up of all three patients.

## Discussion

The metastatic tumour to the thyroid gland is a rare malignant tumour of highly aggressive, commonly occurring in primary malignant tumours of kidney, gastrointestinal system, lung and breast.^1^–^4^ As the above mentioned primary malignant tumours occur mostly in elderly population^9^, also the metastatic malignancy to the thyroid gland is observed in the elderly individuals. They are in their sixth and seventh decades of life and the mean age of our patients were also approximately 60 years similar to that described in the literature.^10^,^11^

An objective estimate of the incidence of the clinical metastasis in the thyroid comes from recent series related with the fine-needle aspiration biopsy (FNAB) performed for tumours of thyroid, which report incidences of up to 7.5%.^12^,^13^ FNAB revealed malignant cytologic features in two of the three patients, but interpreted as only malignancy without any additional comment related with primary or metastatic. The overall experience from series of the patients with metastases to the thyroid gland is variable, but a long-term survival after the surgical intervention to the thyroid is not reported frequently in the literature.^14^–^16^

The clinical history and the pathological diagnosis of the primary site malignancy largely influence the route of histopathologic diagnosis. In our first case, a definitive diagnosis requires absolutely the finding of positivity for CD10 and/or renal cell carcinoma antigen and negativity for thyroglobulin and TTF-1 in areas of clear cell carcinoma in the thyroid in order to differentiate the metastatic clear cell carcinoma of kidney from the primary clear cell malignancy from thyroid.

As in our second case, if patient has a history of primary squamous cell carcinoma of the lung, the differential diagnosis of squamoid malignancy located in the thyroid should be easy. The infiltrative pattern of the tumour in addition to disseminated tumour emboli in lymphatic vessels also should be helpful findings for the metastasis. Negativity of TTF-1 and thyroglobulin of tumour cells might be helpful in metastatic squamous cell carcinoma in the differentiation from the primary squamous cell carcinoma of the thyroid that reported exceptionally.^17^,^18^

There is no very characteristic feature of the metastatic breast carcinoma especially lobular malignancy. To the best of our knowledge, a very few reports described immunohistochemical staining properties. In ductal type carcinoma, the presence of carcinoma containing comedonecrosis area should raise the possibility of the metastatic origin especially from the breast. Also, in addition to positivity of estrogen, progesteron, cerbB-2, gross cystic disease fluid protein-15 (GCDFP-15) and negativity of thyroglobulin, TTF-1, CEA and calcitonin will make possible the differential diagnosis of tumours originated from the breast. In our case, CK7 positivity and estrogen positivity were presented and markers like thyroglobulin, TTF-1, CEA and calcitonin were negative.

The management and the extent of surgery in metastatic involvement of the thyroid gland have not been firmly determined by a uniform international consensus. The management depends on the primary site of the original tumour, the presence of other metastases and symptoms caused by the thyroid mass. Generally, patients with the thyroid metastasis who have already demonstrated metastases to organs other than the thyroid gland had a poor prognosis.

On the other hand, in cases with the solitary thyroid metastasis, there have been several case reports showing that a long term survival is possible after thyroidectomy. The metastasis operation may improve a prognosis in certain cases also in other patients with the advance oncological disease.^19^ Therefore, a surgical resection is regarded as the best treatment for a metastatic tumour, especially, if isolated, the solitary thyroid metastasis was found. There is also still a doubt about the extent operation in thyroid surgery because of the complications after total thyroidectomy.^20^ If the metastasis is known preoperatively, lobectomy and isthmectomy are enough but when the diagnosis is doubtful or multifoci of metastasis are found, total tyhroidectomy is the choice of the surgical treatment.

There is also the possibility of metastases to cervical lymph nodes, but there is no data that determinate the importance of the lymph node dissection. However, when the diagnosis is established postoperatively only, based on the histopathological examination of the surgical specimen, a secondary radical procedure after previously performed subtotal thyroidectomy seems unwarranted. As the adjuvant treatment, chemotherapy and radiotherapy could be useful according to the type of primary cancer that metastases to the thyroid. Also the interferon treatment may be useful for metastatic clear cell cancer of kidney.^10^,^21^,^22^ In cases with an anaesthetic risk or co-morbid condition that preclude surgery, the radiotherapy should be considered.

In our three cases, FNAB only shows suspicious of malignant cytology and the type of surgery is total thyroidectomy in two cases and bilateral near total thyroidectomy in one patient who accepted useful surgery for the malignant disease of the thyroid gland. Postoperatively, we could not use adjuvant chemotherapy since the patients refused it.

In conclusion, the occurrence of metastatic malignancy to the thyroid is very unusual. It should be considered in differential diagnosis of an atypical histological appearance of the unexpected course of the tumour of the thyroid. Very careful histopathological examination and immunohistochemical panel antibody study of the lesion are necessary to confirm the diagnosis. It is important to exclude a systemic dissemination of any primary tumour located elsewhere and the prognosis in a patient with metastatic malignancy located in the thyroid is poor. The appearance of the unusual histological type of tumour in thyroid should be considered seriously and warrants a detailed immunohistochemical panel. Metastases resection is the choice of the treatment in a case of the isolated solitary metastasis.

## Figures and Tables

**FIGURE 1A.**
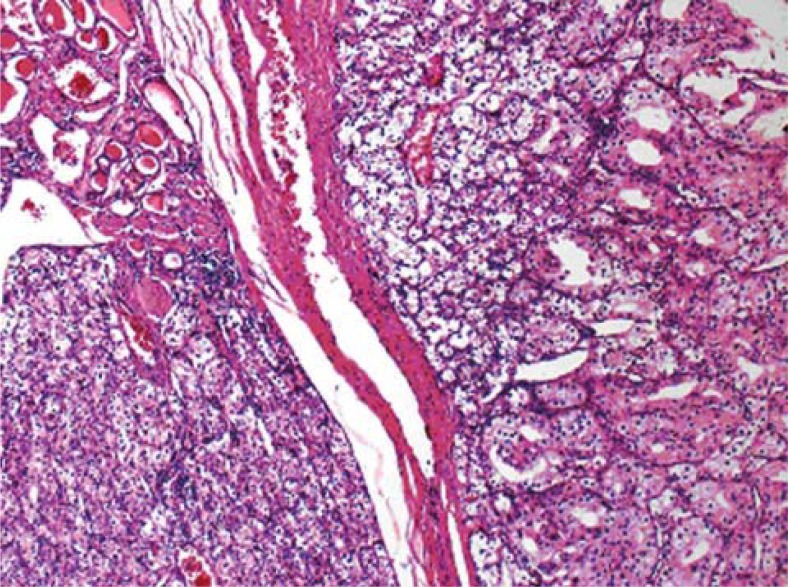
Large, two solid tumour islands separated with fibrous septum and normal thyroid acini (upper left) were seen (HE-X40).

**FIGURE 1B.**
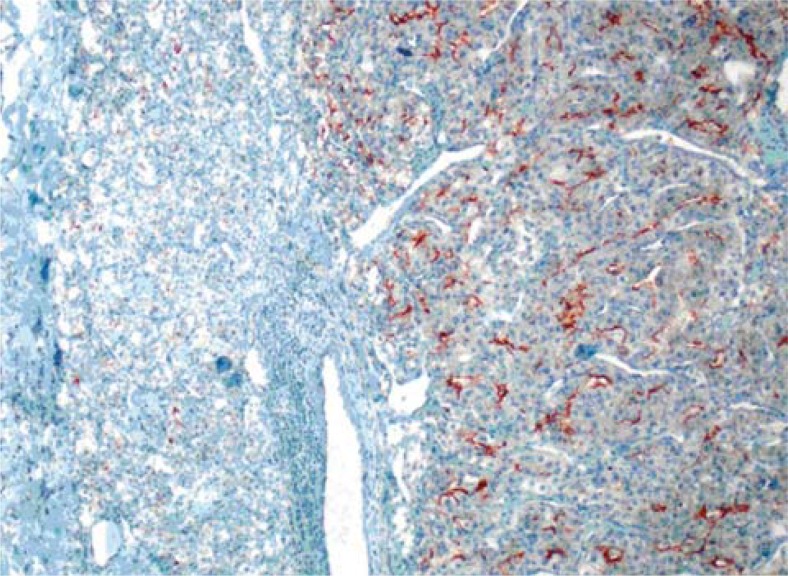
Diffuse, strong CD10 positivity were seen in neoplastic areas especially located in right part of the image (X100).

**FIGURE 1C.**
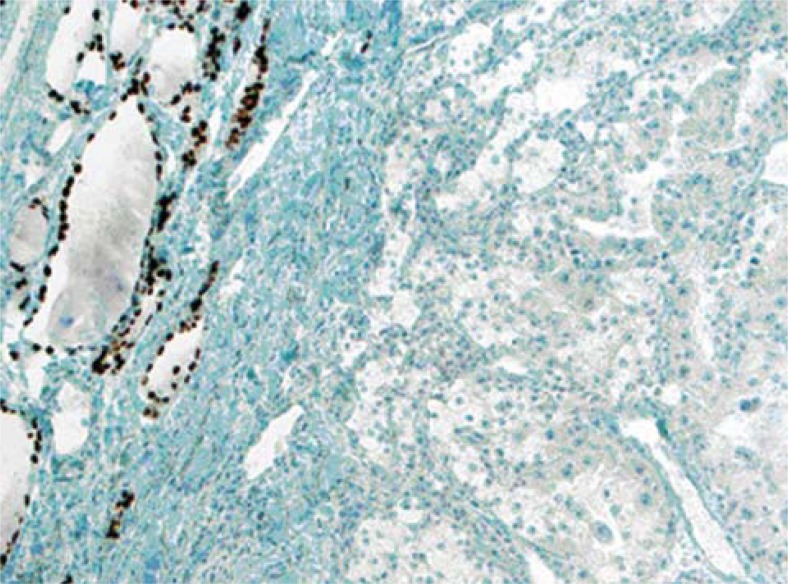
TTF-1 positivity was seen in residual thyroid follicles located in left part of the image (X100).

**FIGURE 3A.**
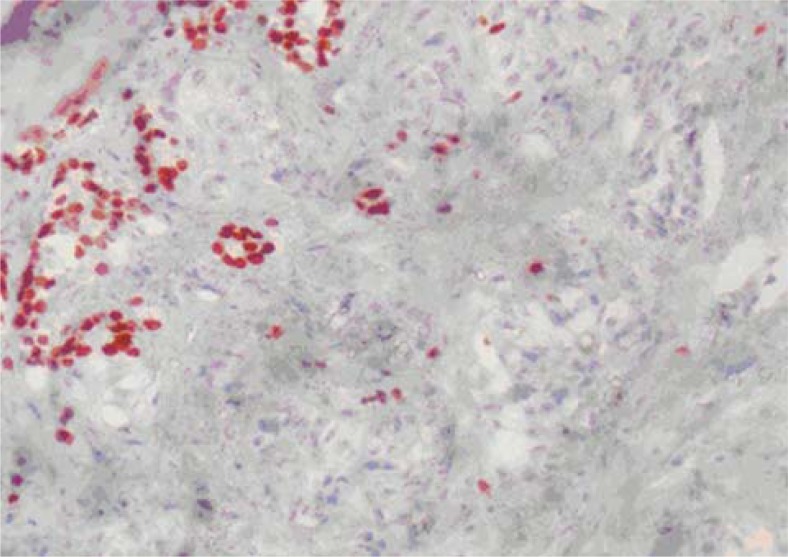
Small, ductuli like tumour cells, residual thyroid follicles and pleomorphic tumour cells in high magnification and necrosis were seen (inset) (HE-X400).

**FIGURE 3B.**
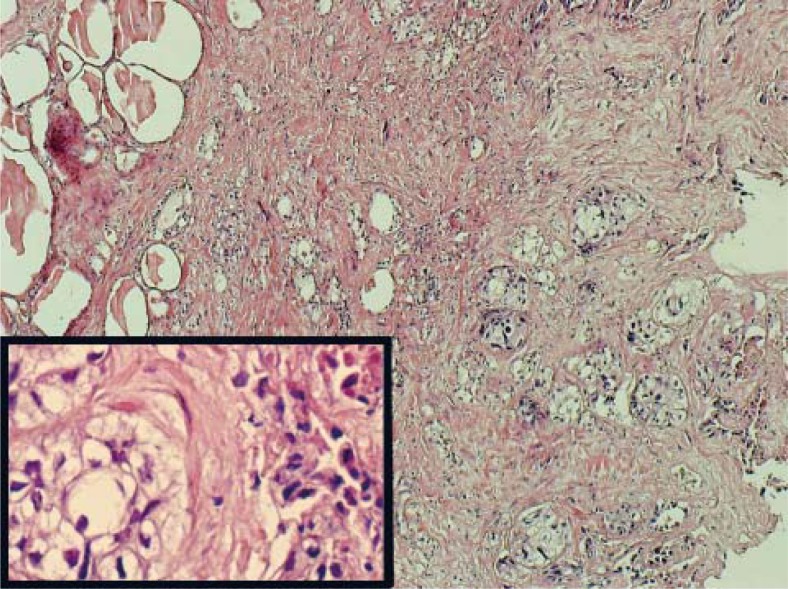
TTF-1 nuclear immunreactivity were seen in residual thyroid follicles located in upper left corner of the image whereas tumour cells were negative in case 3 (X100).

**TABLE 1.** General characteristics of the patients

	**Age**	**Gender**	**Primary tumour surgery**	**Primary tumour diagnosis**	**Metastasis in thyroid**
**Case 1**	54	Male	Nephrectomy	Kidney renal cell carcinoma	Bilateral
**Case 2**	63	Male	Lung lobectomy	Lung squamous cell carcinoma	Unilateral right
**Case 3**	43	Female	Mastectomy+ axillary dissection	Breast ductal carcinoma	Unilateral left

## References

[1] Willis RA (1931). Metastatic tumors in the thyroid gland. Am J Pathol.

[2] Abrams HL, Spiro R, Goldstein N (1950). Metastases in carcinoma: analysis of 1,000 autopsy cases. Cancer.

[3] Shimaoka K, Sokal J, Pickrea J (1962). Metastatic neoplasms in the thyroid gland. Cancer.

[4] Silverberg SG, Vidone RA (1966). Metastatic tumors in the thyroid. Pacif Med Surg.

[5] Boggess MA, Hester TO, Archer SM (1996). Renal clear cell carcinoma appearing as a left neck mass. Ear Nose Throat J.

[6] Berge T, Lundberg S (1977). Cancer in Malmo 1958–1969. An autopsy study. Acta Pathol Microbiol Scand.

[7] Chen H, Nicol TL, Udelsman R (1999). Clinically significant, isolated metastatic disease to the thyroid gland. World J Surg.

[8] Chung SY, Kim EK, Kim JH, Oh KK, Kim DJ, Lee YH (2001). Sonographic findings of metastatic disease to the thyroid. Yonsei Med J.

[9] Debevec L, Jeric T, Kovac V, Bitenc M, Sok M (2009). Is there any progress in routine management of lung cancer patients? A comparative analysis of an institution in 1996 and 2006. Radiol Oncol.

[10] Heffess CS, Wenig BM, Thompson LD (2002). Metastatic renal cell carcinoma to the thyroid gland: a clinicopathologic study of 36 cases. Cancer.

[11] Cichoń S, Anielski R, Konturek A, Barczyński M, Cichoń W (2006). Metastases to the thyroid gland: seventeen cases operated on in a single clinical center. Langenbecks Arch Surg.

[12] Michelow PM, Leiman G (1995). Metastases to the thyroid gland: diagnosis by aspiration cytology. Diagn Cytopathol.

[13] Watts NB (1987). Carcinoma metastatic to the thyroid: prevalence and diagnosis by fine-needle aspiration cytology. Am J Med Sci.

[14] McCabe DP, Farrar WB, Petkov TM, Finkelmeier W, O’Dwyer P, James A (1985). Clinical and pathologic correlations in disease metastatic to the thyroid gland. Am J Surg.

[15] Nakhjavani MK, Gharib H, Goellner JR, van Heerden JA (1997). Metastases to the thyroid gland: a report of 43 cases. Cancer.

[16] Freund HR (1965). Surgical treatment of metastases to the thyroid gland from other primary malignancies. Ann Surg.

[17] Long JL, Strocker AM, Wang MB, Blackwell KE (2009). EGFR expression in primary squamous cell carcinoma of the thyroid. Laryngoscope.

[18] Eom TI, Koo BY, Kim BS (2008). Coexistence of primary squamous cell carcinoma of thyroid with classic papillary thyroid carcinoma. Pathol Int.

[19] Ocvirk J (2009). Advances in the treatment of metastatic colorectal carcinoma. Radiol Oncol.

[20] Besic N, Zagar S, Pilko G, Peric B, Hocevar M (2008). Influence of magnesium sulphate infusion before total thyroidectomy on transient hypocalcemia – a randomised study. Radiol Oncol.

[21] Wood K, Vini L, Harmer C (2004). Metastases to the thyroid gland: the Royal Marsden experience. EJSO.

[22] Lesalnieks I, Winter H, Bareck E, Sotiropoulos GC, Goretzki PE, Klinkhammer-Schalke M (2008). Thyroid metastases of renal cell carcinoma: clinical course in 45 patients undergoing surgery. Assesment of factors affecting patients survival. Thyroid.

